# Oral Health-Related Quality of Life and Its Determinants in Children and Adolescents with Autism Spectrum Disorder: A Scoping Review

**DOI:** 10.3390/dj14080492

**Published:** 2026-08-06

**Authors:** Alice Murariu, Livia Bobu, Gianina Iovan, Gabriela Luminița Gelețu, Laura Ioana Leon, Alexandra Cornelia Teodorescu, Diana Zapodeanu, Bianca-Andreea Onofrei, Dragoș Nicolae Frățilă, Costin Iulian Lupu, Elena-Raluca Baciu

**Affiliations:** Grigore T. Popa University of Medicine and Pharmacy Iasi, 700115 Iasi, Romania; alice.murariu@umfiasi.ro (A.M.); gianina.iovan@umfiasi.ro (G.I.); gabriela.geletu@umfiasi.ro (G.L.G.); laura.leon@umfiasi.ro (L.I.L.); cornelia.teodorescu@umfiasi.ro (A.C.T.); diana-zapodeanu@umfiasi.ro (D.Z.); bianca.onofrei@umfiasi.ro (B.-A.O.); dragos.fratila@umfiasi.ro (D.N.F.); iulian.lupu@umfiasi.ro (C.I.L.); elena.baciu@umfiasi.ro (E.-R.B.)

**Keywords:** autism spectrum disorder, children, adolescents, oral health, quality of life

## Abstract

**Background/Objectives**: Individuals with autism spectrum disorder (ASD) represent a special population whose characteristics may adversely affect both their own and their families’ quality of life. These characteristics include poor oral health, sensory hypersensitivity, restrictive and repetitive behaviours, communication difficulties, medication-related adverse effects, and associated comorbidities. This scoping review aimed to evaluate the oral health-related quality of life (OHRQoL) of children and adolescents with ASD, as perceived by their parents/caregivers, and to identify the factors associated with these outcomes. **Methods**: Literature searches were conducted in the MEDLINE/PubMed, Scopus, Web of Science, Embase, and Google Scholar databases. Studies published between 2016 and May 2026 were considered for inclusion. **Results**: Of the 799 records identified, 23 studies met the eligibility criteria. Among these, 15 used the Parental-Caregiver Perceptions Questionnaire (P-CPQ), an instrument specifically developed for children with cognitive impairments. Most studies reported statistically significant associations between poorer OHRQoL and dental caries experience, lower household income, older age, male sex, and lower parental oral health literacy. Conversely, preventive interventions and comprehensive dental rehabilitation performed under general anaesthesia were associated with improvements in the quality of life of both children and their families. Parents of children with ASD also reported a greater emotional burden, primarily related to responsibility for toothbrushing, dental attendance, the child’s general health status, and the family’s financial situation. **Conclusions**: The majority of the included studies indicate that, according to parental reports, children and adolescents with ASD experience poor OHRQoL, particularly in the domains of emotional well-being, social well-being, functional limitations, and oral symptoms. These findings highlight the need for targeted preventive strategies and multidisciplinary interventions aimed at improving both oral health and overall quality of life in this vulnerable population.

## 1. Introduction

Autism spectrum disorder (ASD) is a common neurodevelopmental disorder characterised by persistent difficulties in social interaction and communication, alongside restricted and repetitive patterns of behaviour. It frequently co-occurs with a range of other medical and psychiatric conditions [1,2,3]. According to the World Health Organization (WHO), approximately one in every 127 individuals is affected by ASD worldwide [4]. In Europe, the reported prevalence varies considerably, ranging from 0.4% in Lithuania to 14.3% in Romania, with a prevalence approximately 3.5 times higher in males than in females [5]. Furthermore, data released by the Centers for Disease Control and Prevention (CDC) in 2025 indicate a substantial increase in ASD prevalence over recent decades, rising from approximately 1 in 150 children in 2000 to 1 in 31 in 2022 [6].

Clinical features of ASD can usually be identified during early childhood, with many characteristic symptoms becoming apparent around the age of two. Symptom severity varies from mild to severe and may change over time, while some manifestations may be masked through complex compensatory mechanisms [7].

People with ASD often experience difficulties interpreting both verbal and non-verbal communication, including gestures, facial expressions, and tone of voice. They commonly display repetitive and restrictive behaviours, and even minor changes to established routines may be perceived as highly distressing, frequently triggering anxiety. Another hallmark of ASD is atypical sensory processing, manifested as either hypersensitivity or hyposensitivity to auditory, tactile, gustatory, olfactory, visual, thermal, and painful stimuli [8,9,10].

These sensory processing differences can make the dental environment particularly challenging. Bright clinical lighting, unexpected sounds, unpleasant smells and tastes, and tactile sensations associated with dental equipment and oral hygiene procedures may all provoke discomfort and distress during dental treatment [11].

Anxiety is highly prevalent among individuals with ASD, particularly in unfamiliar social situations or when faced with changes to routine. Anxiety may negatively affect both psychological and physical well-being, thereby reducing the quality of life of affected individuals and their families [12].

More than 60% of children with ASD engage in self-injurious behaviours, including severe self-biting, pinching, and lip biting, thereby increasing their susceptibility to periodontal disease, dental trauma, and other oral disorders [13,14,15,16].

Evidence consistently demonstrates that oral health is considerably poorer among individuals with ASD than among their neurotypical peers. Numerous studies have reported a higher prevalence of dental caries, periodontal diseases, dental trauma, dental pain, and halitosis in both children and adults with ASD [17,18,19,20,21,22,23,24,25].

Poor oral hygiene, often resulting from refusal to perform toothbrushing independently or the need for parental assistance, further compromises quality of life by increasing the risk of dental caries, periodontal disease, pain, and nutritional deficiencies [26,27,28,29].

Parents and caregivers frequently prioritise the neuropsychiatric aspects of ASD while unintentionally overlooking oral health problems. Consequently, oral conditions are often addressed only when they become emergencies, thereby limiting the effectiveness of preventive and early therapeutic interventions [30,31]. Furthermore, parents of children with ASD experience significantly higher levels of stress than parents of typically developing children. Behavioural problems are strongly associated with increased parental stress and represent important risk factors for parental anxiety and depression [32,33]. 

Oral health-related quality of life (OHRQoL) is a multidimensional construct that evaluates the impact of oral conditions on physical comfort, emotional well-being, self-esteem, and social functioning [34,35,36]. 

Among individuals with ASD, numerous factors contribute to impaired OHRQoL, including poor oral health, behavioural disturbances, barriers to accessing dental care, and socioeconomic disadvantage [37]. Understanding parental perceptions regarding the oral health of children with ASD is therefore essential for developing effective preventive strategies and improving both oral health outcomes and quality of life in this population [38].

Although numerous studies have investigated the relationship between oral health status and OHRQoL among children with ASD, the available evidence remains heterogeneous and inconclusive. Likewise, the influence of socioeconomic determinants and preventive interventions has not yet been comprehensively outlined. Therefore, the aim of the present scoping review was to synthesise the available evidence regarding parent- and caregiver-reported OHRQoL in children and adolescents with ASD by examining studies published between 2016 and May 2026. A scoping review methodology was considered the most appropriate approach because all included studies employed cross-sectional designs, and considerable heterogeneity existed in the OHRQoL assessment instruments used, precluding the conduct of a systematic review and meta-analysis.

## 2. Materials and Methods

This scoping review was conducted in accordance with the methodology described by Aromataris et al. in the JBI Manual for Evidence Synthesis [39] and followed the PRISMA Extension for Scoping Reviews (Table S1) developed by Tricco et al. [40] to ensure a comprehensive and systematic approach.

The review process comprised the following stages:

1. Formulation of the research questions.
2. Identification of relevant studies addressing the predefined research questions.
3. Selection of eligible studies according to predetermined inclusion and exclusion criteria.
4. Development of the review database.
5. Data extraction, synthesis, and reporting of the findings.

### 2.1. Review Questions

This scoping review was guided by the following research questions:

1. Is the OHRQoL of children and adolescents with ASD poorer than that of their neurotypical peers?
2. What are parents’ and caregivers’ perceptions of the oral health and OHRQoL of children with ASD?
3. How do oral health status and socio-demographic and socioeconomic factors influence the different domains of OHRQoL in children and adolescents with ASD?

These questions were formulated to guide the review process and address the objectives of the present study.

### 2.2. Information Sources and Search Strategy

A systematic literature search was conducted in the following electronic databases: MEDLINE/PubMed, Scopus, Web of Science, Embase, and Google Scholar. Studies published between January 2016 and May 2026 were considered eligible for inclusion.

The search strategy combined the following keywords: “oral health”, “oral health-related quality of life”, “autism spectrum disorder”, “autism”, “autistic disorder”, “children”, “adolescents”, and “parental perception”. These terms were combined using the Boolean operators AND and OR to optimise the sensitivity and specificity of the search and to identify all potentially relevant studies across the selected databases.

The detailed search strategies used for each database are presented in Table 1.

### 2.3. Eligibility Criteria

To ensure the quality and relevance of the studies included in this scoping review, predefined inclusion and exclusion criteria were established (Table 2).

The inclusion criteria comprised full-text articles published in English between January 2016 and May 2026 that investigated OHRQoL of children and adolescents with ASD. Eligible studies examined OHRQoL from the perspectives of parents or caregivers and/or evaluated its relationship with clinical oral health status and dental interventions.

Studies were excluded if they were published before 2016, were available only as conference abstracts, were published in languages other than English, were inaccessible in full text (including those not available through institutional access), or were duplicates. In addition, systematic reviews, narrative reviews, scoping reviews, and studies that did not address the predefined review objective—namely the assessment of OHRQoL in children and adolescents with ASD—were excluded.

### 2.4. Study Selection and Data Extraction

The study selection process was conducted in two stages. During the first stage, the titles and abstracts of the retrieved records were screened for relevance. In the second stage, the full texts of potentially eligible studies were independently assessed by three reviewers (A.M., S.S., and L.B.) against the predefined inclusion and exclusion criteria. Any disagreements between the reviewers were resolved through consultation with a fourth reviewer (E.-R.B.).

To ensure comprehensive and consistent data collection, information extracted from the included studies was recorded using a standardised data extraction form specifically developed for this review. The extracted variables are summarised in Table 3.

The synthesis of the extracted data aimed to provide a comprehensive overview of the impact of oral diseases and oral conditions on the OHRQoL of children and adolescents with ASD. It also aimed to provide an overview of parents’ and caregivers’ perspectives regarding oral health literacy and their satisfaction with the quality of life of both their children and their families. Furthermore, the review sought to identify gaps and inconsistencies in the current evidence, acknowledge the limitations of the existing literature, and highlight priorities for future research.

## 3. Results

The literature search was conducted between May and June 2026. A total of 799 records were identified across five electronic databases: MEDLINE/PubMed (*n* = 144), Scopus (*n* = 82), Web of Science (*n* = 131), Embase (*n* = 110), and Google Scholar (*n* = 332).

Of these, 122 records were excluded because they were duplicates, conference abstracts, review articles, published outside the predefined study period, or unavailable through open access or institutional subscription. The remaining 677 records underwent title and abstract screening, during which 424 were excluded as irrelevant. Consequently, 253 full-text articles were assessed for eligibility.

Following full-text assessment, 230 studies were excluded for the following reasons: they did not focus on OHRQoL (*n* = 112), did not investigate autism spectrum disorder (ASD) (*n* = 32), or primarily addressed other oral health topics, including oral health interventions, oral health status, or dental procedures (*n* = 86).

Ultimately, 23 studies met the eligibility criteria and were included in the qualitative synthesis.

**Figure 1:**
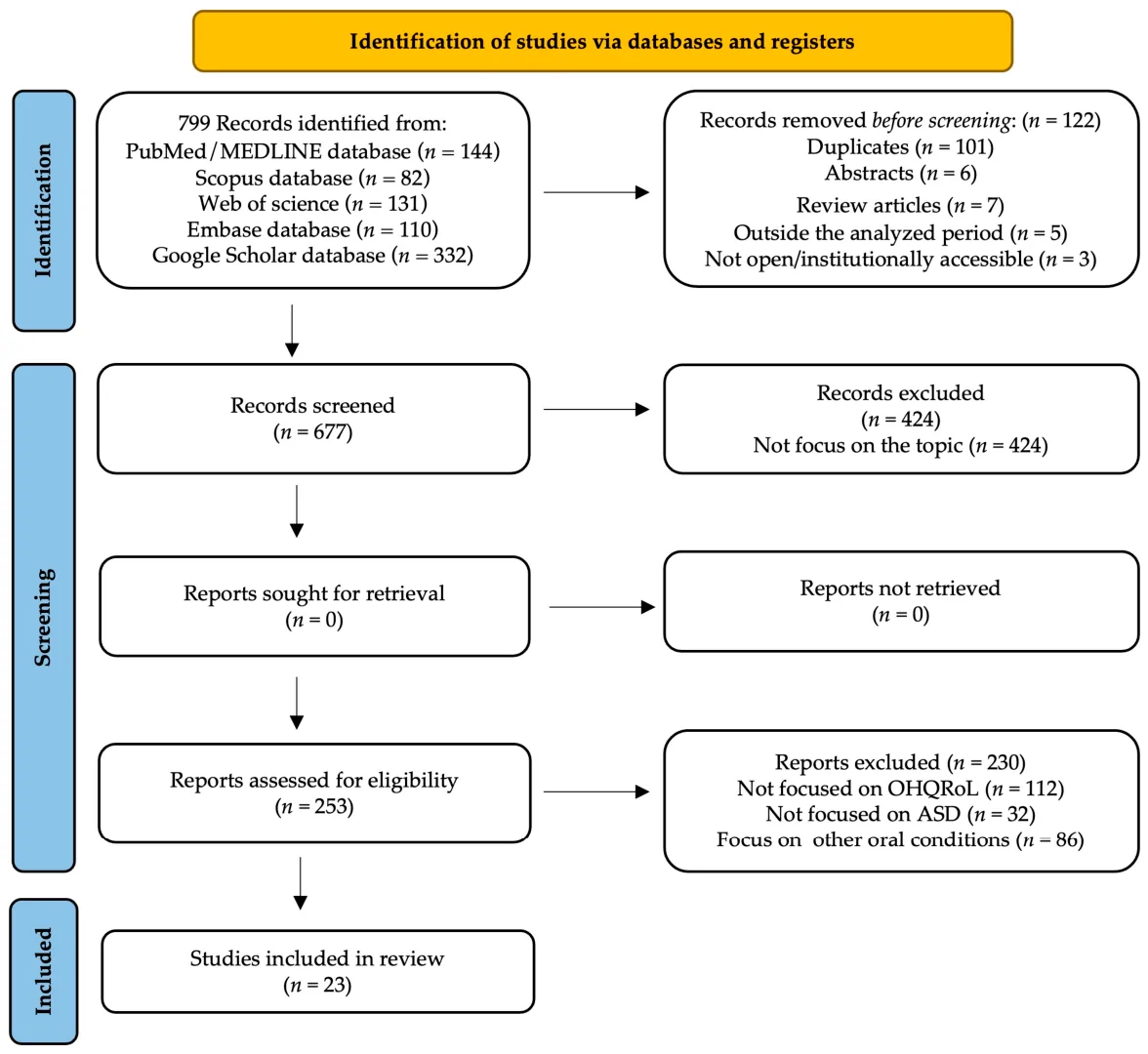
The flow diagram for the identification, screening and eligibility of studies (PRISMA-Scr).

The results of the literature search and study selection process are summarised in the PRISMA Extension for Scoping Reviews (PRISMA-ScR) flow diagram presented in Figure 1 [40].

### 3.1. General Characteristics of the Included Studies

A total of 23 studies published between January 2016 and May 2026 met the eligibility criteria and were included in this review. The highest number of publications was recorded in 2024 (*n* = 5), followed by 2026, 2025 and 2020 (*n* = 3 each), 2023, 2022 and 2021 (*n* = 2 each), 2019, 2018 and 2018 (*n* = 1 each).

**Figure 2:**
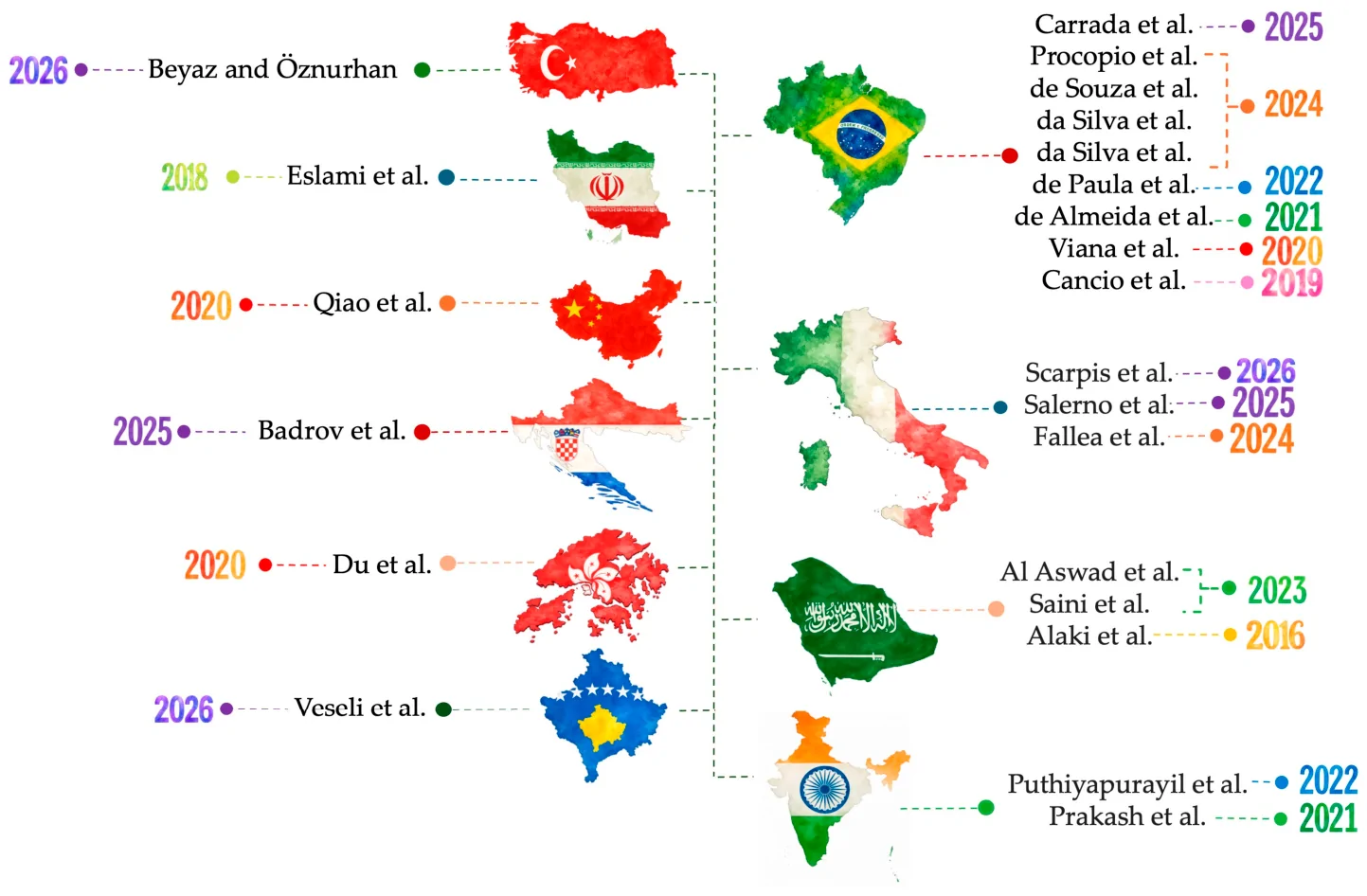
Geographic distribution and publication timeline of the included studies assessing oral health-related quality of life in children and adolescents with autism spectrum disorder [41,42,43,44,45,46,47,48,49,50,51,52,53,54,55,56,57,58,59,60,61,62,63].

Regarding study design, the vast majority of the included studies (91.3%, *n* = 21) were cross-sectional surveys, while one study employed a case-control design and one was a prospective longitudinal study. The studies were conducted across a range of countries. Brazil contributed the largest number of publications (*n* = 9), followed by Italy (*n* = 3), Saudi Arabia (*n* = 3), and India (*n* = 2). One study each originated from China, Croatia, Hong Kong, Iran, Kosovo, and Türkiye (Figure 2).

The general characteristics of the included studies are summarised in Table 4, which presents the following information: first author, country and year of publication, study design, questionnaire used, principal findings, and conclusions.

**Table 4:** Main characteristics of the included studies.

First Author, Country, Year	Aim	Study Design	Sample	Questionnaire	Results	**Conclusions**
Alaki et al., Saudi Arabia, 2016 [41]	To evaluate parents’ perceptions of OHRQoL and oral health status in children with ASD compared with healthy children.	Comparative cross-sectional study	ASD group: 75 parents and their children with ASD, aged 6–12 years; Control group: 99 parents and their healthy children.	Franciscan Hospital for Children Oral Health-Related Quality of Life Questionnaire (FHC-OHRQoL)	Parents of children with ASD reported significantly more problems affecting daily life and greater parental concerns (*p* = 0.004 and *p* = 0.008, respectively). Children with ASD also had significantly poorer oral health status scores. Furthermore, they presented a higher prevalence of extraoral lesions (traumatic injuries or scars) and intraoral lesions (*p* < 0.001 for both), a higher prevalence of dental caries (*p* = 0.013), and greater caries severity (*p* = 0.003) than the control group.	Parents of children with ASD perceived significantly poorer oral health status and OHRQoL compared with parents of healthy children.
Al Aswad et al., Saudi Arabia, 2023 [42]	To compare parents’ perceptions of OHRQoL in children with and without ASD following comprehensive oral rehabilitation under general anaesthesia.	Comparative cross-sectional study	ASD group: 114 children with ASD, aged 4–9 years. Control group: 116 children without ASD, aged 4–9 years.	Parental-Caregiver Perceptions Questionnaire (P-CPQ); Family Impact Scale (FIS)	Depending on the assessed domain, children with ASD had lower scores for functional limitations and emotional well-being but higher scores for oral symptoms and social well-being than children without ASD. A statistically significant difference between the two groups was observed only for the parental emotions domain (*p* < 0.05).	Both groups achieved comparable P-CPQ scores, suggesting that comprehensive oral rehabilitation performed under general anaesthesia had a positive impact on oral health-related quality of life in both children with ASD and their neurotypical peers.
Badrovet al., 2025, Croatia [43]	To investigate the impact of oral health status on the OHRQoL of children with ASD and their families.	Cross-sectional study	121 parents of children with ASD, aged 3–18 years.	P-CPQ; FIS; a 70-item self-administered questionnaire assessing socio-demographic characteristics, parents’ perceptions of their child’s oral health, oral hygiene practices, and access to dental care	The prevalence of dental caries was 48.8%, bruxism 38.8%, and dental pain 28.8%. Within the P-CPQ-16, the highest mean score (5.84) was recorded for the emotional well-being domain. Within the FIS-8, the highest mean score (5.16) was observed for the parental activities domain.	Parents’ socio-demographic characteristics did not significantly influence P-CPQ-16 or FIS-8 scores. Poor oral health in children with ASD was associated with poorer OHRQoL for both the children and their families. Uncooperative behaviour, inadequate access to dental care, and the need for treatment under general anaesthesia were strongly associated with poorer OHRQoL scores.
Beyaz and Öznurhan, Türkiye, 2026 [44]	To compare parental oral health literacy and OHRQoL between children with ASD and neurotypical children.	Comparative cross-sectional study	142 children aged 3–15 years: 72 with ASD and 70 in the control group.	Oral Health Literacy Assessment Task for Pediatric Dentistry (TOHLAT-P); Pediatric Oral Health-Related Quality of Life Measure (POQL)	Parents of children in the control group achieved significantly higher oral health literacy scores than parents of children with ASD. No statistically significant differences were observed between the groups in the physical functioning and emotional functioning domains. However, significant differences were found in the social functioning domain and in the overall POQL score.	Parental oral health literacy was significantly associated with the OHRQoL of children with ASD.
Cancio et al., Brazil, 2019 [45]	To evaluate OHRQoL of children and adolescents with ASD.	Cross-sectional study	70 children with ASD, aged 2–14 years, together with their parents/caregivers.	P-CPQ	Impact scores were significantly higher among male participants than females in the oral symptoms domain (7.50 vs. 6.43) and among children with caries experience compared with those without caries experience in the social well-being domain (8.00 vs. 3.14) (*p* < 0.05).	The findings suggest a greater impact on OHRQoL among boys in the oral symptoms domain and among children with ASD with caries experience in the social well-being domain.
Carrada et al., Brazil, 2025 [46]	To evaluate the association between caregivers’ oral health literacy and OHRQoL of children and adolescents with ASD.	Cross-sectional study	30 children with ASD and their parents/caregivers; mean age 7.8 ± 4.07 years.	Brazilian Version of the Rapid Estimate of Adult Literacy in Dentistry (BREALD-30); P-CPQ-13; FIS	No statistically significant association was found between oral health status and OHRQoL. Families with a monthly income of up to two minimum wages were 2.25 times more likely to report a negative perception of their children’s OHRQoL than families with a higher income. Overall, 43.3% of parents reported a high impact on OHRQoL according to the P-CPQ, while 45.7% reported a high impact according to the FIS.	Household income and parental oral health literacy were significant determinants of parents’ negative perceptions of the OHRQoL of both their children with ASD and their families.
da Silva et al., Brazil, 2024 [47]	To evaluate the influence of socio-demographic characteristics, oral hygiene practices, and the psychosocial need for orthodontic treatment on the emotional well-being of parents of children with and without ASD.	Comparative cross-sectional study	ASD group: 72 parent–child pairs, children aged 6–14 years. Control group: 72 parent–child pairs of neurotypical children.	Parental Emotions domain of the FIS questionnaire.	Parents of children with ASD had significantly higher Parental Emotions scores (*p* < 0.001). Factors associated with higher emotional burden included responsibility for toothbrushing (PR = 1.34; 95% CI: 1.12–1.59), a mandatory need for orthodontic treatment (PR = 1.25; 95% CI: 1.07–1.46), and lower parental educational attainment (PR = 1.45; 95% CI: 1.02–2.07). Parents with lower household income showed a lower prevalence of adverse parental emotions (PR = 0.57; 95% CI: 0.35–0.93).	Parents of children with ASD experienced a greater emotional burden than parents of neurotypical children. Responsibility for toothbrushing, the psychosocial need for orthodontic treatment, and household income were significant factors associated with parental emotional well-being.
da Silva et al., Brazil, 2024 [48]	To evaluate the impact of sociodemographic factors and oral health status on OHRQoL and family functioning in children with and without ASD.	Comparative cross-sectional study	ASD group: 72 children with ASD, aged 6–14 years, and their caregivers. Control group: 72 neurotypical children, aged 6–14 years, and their caregivers.	P-CPQ; FIS	Factors associated with poorer perceived OHRQoL included older child age, lower caregiver educational attainment, higher IOTN scores, bruxism (PR = 1.20; 95% CI: 1.01–1.41), and lip sucking. Lower parental educational attainment (PR = 1.75; 95% CI: 1.10–2.80) and higher caregiver-perceived IOTN scores were also associated with a greater negative impact on family functioning.	Caregivers of children with ASD perceived poorer OHRQoL, and families in the ASD group were more adversely affected by sociodemographic factors and children’s oral health status.
de Almeida et al., Brazil, 2021 [49]	To evaluate the impact of dental treatment on OHRQoL of children and adolescents with ASD from the parents’ perspective.	Prospective longitudinal study	115 children and adolescents with ASD, aged 6–14 years	P-CPQ	Parents completed the questionnaire before dental treatment and three months after treatment. The interventions included restorative, periodontal, endodontic, and surgical procedures. The mean P-CPQ score decreased significantly from 13.2 ± 6.4 at baseline to 3.4 ± 2.2 three months after treatment (*p* < 0.001).	From the parents’ perspective, dental treatment resulted in a significant improvement in the OHRQoL of children and adolescents with ASD.
de Paula et al., Brazil, 2022 [50]	To evaluate the impact of dental treatment on OHRQoL in children with ASD	Cross-sectional study	27 randomly selected children with ASD, aged 4–14 years.	Brazilian Early Childhood Oral Health Impact Scale (B-ECOHIS)	OHRQoL was assessed before dental treatment and 14 days after treatment. Dental interventions included preventive procedures (fluoride varnish application and fissure sealants) and restorative treatment using composite resin and glass ionomer cement. Total B-ECOHIS scores and all domain scores decreased significantly following treatment, indicating an improvement in the OHRQoL of both the children and their families.	Total B-ECOHIS scores and scores across all domains decreased significantly following treatment, indicating an improvement in the OHRQoL of both children with ASD and their families.
de Souza et al., Brazil, 2024 [51]	To evaluate OHRQoL in children and adolescents with ASD through parents’ and caregivers’ perceptions	Cross-sectional study	51 children and adolescents aged 4–18 years attending an ASD centre	PCP-Q; FIS	The overall PCP-Q score ranged from 0 to 25, with a mean score of 7.41 (±6.64). PCP-Q scores were significantly higher among participants with dental caries experience in the primary dentition (*p* < 0.05). No significant differences in OHRQoL scores were observed according to family income or the mother’s educational level.	According to parents’ perceptions, children and adolescents with ASD showed a greater impact in the functional limitations domain of the PCP-Q. Dental caries experience in the primary dentition was associated with higher mean scores across all PCP-Q domains.
Du et al., Hong Kong, 2020 [52]	To evaluate HRQoL and OHRQoL in preschool children with ASD, based on parents’ perceptions.	Case-control study	Test group: parents of 253 children with ASD, aged 2–6 years; Control group: parents of 257 typically developing preschool children, aged 2–6 years.	Pediatric Quality-of-Life Inventory Version 4.0 (PedsQL™ 4.0) Early Childhood Oral Health Impact Scale (ECOHIS)	HRQoL was positively correlated with OHRQoL. The risk of poor OHRQoL was four times higher among children with a history of dental visits (*p* < 0.001) and 3.2 times higher in relation to the child’s medical condition (*p* < 0.001).	The OHRQoL of children with ASD was poorer than that of children without ASD.
Eslami et al., Iran, 2018 [53]	To evaluate parents’ perceptions of their families’ OHRQoL in relation to their child’s oral health status.	Comparative cross-sectional study	Test group: *n* = 70 families with at least one child with ASD. Control group: *n* = 70 families with typically developing children.	PedsQL oral health scale questionnaire	A significant difference was observed in the total PedsQL score between children with ASD and typically developing children (425 vs. 375, respectively). Parents of typically developing children reported more oral health problems (*p* < 0.001). However, children with ASD had significantly more difficulties in the social (*p* = 0.027) and communication (*p* = 0.004) domains compared with the typically group.	According to parents’ perceptions, the OHRQoL of children with ASD was better than that of typically developing children. Nevertheless, parents of children with ASD reported greater difficulties in the social and communication domains.
Fallea et al., Italy, 2024 [54]	To evaluate the impact of OHRQoL of children and adolescents with ASD.	Cross-sectional study	163 children and adolescents with ASD, aged: 7–8 years	Oral Health Assessment Tool (OHAT); EuroQol 5-Dimensions Youth version (EQ-5D-Y)	A positive association was found between oral health status, as assessed by the OHAT, and quality of life, as measured by the EQ-5D-Y (*p* = 0.00271). A negative association was observed between sialorrhea and EQ-5D-Y scores. A positive association was also identified between OHAT scores and the autonomy subscale of the EQ-5D-Y.	Older children demonstrated poorer quality of life, and the impact on their families was also more pronounced.
Prakash et al., India, 2021 [55]	To evaluate oral health status and parents’ perceptions of the oral health of children with ASD.	Observational study	300 parents with ASD children	A questionnaire with seven items	Only 18.33% of parents were aware that oral health can influence overall health. A total of 59.1% reported visiting the dentist only when their child experienced pain, while 52% reported experiencing constant stress. Dental caries were present in 75% of children with ASD, although the mean oral hygiene index indicated generally good oral hygiene.	Autism is a condition that affects both general physical health and oral health. As parents are directly involved in caring for their children, they also experience a reduced quality of life.
Procopio et al., Brazil, 2024 [56]	To evaluate parents’ and caregivers’ perceptions of the impact of oral conditions on OHRQoL of children and adolescents with ASD compared with their typically developing peers.	Comparative cross-sectional study	Test group: *n* = 80 children/adolescents with ASD, aged 3–16 years, and their parents. Control group: *n* = 80 typically developing children/adolescents, aged 3–16 years, and their parents.	P-CPQ	No significant differences were found between the groups in the overall P-CPQ score or in any of the domain scores (oral symptoms, functional limitations, and well-being) (*p* > 0.05). Dental caries experience was associated with a greater negative impact on the quality of life of children and adolescents with ASD (*p* < 0.05).	Dental caries experience may negatively affect the OHRQoL of children and adolescents with ASD.
Puthiyapurayil et al., India, 2022 [57]	To evaluate OHRQoL of children with intellectual disabilities and barriers to accessing oral healthcare.	Cross-sectional study	300 children with intellectual disabilities, including 69 children with ASD, aged 4–12 years.	P-CPQ	During the clinical examination, dental status was assessed using the DMFT and dmft indices. Caries severity was classified into categories ranging from 0 (caries-free) to ≥5 (high severity). Children with ASD had the highest P-CPQ scores across all domains compared with children with other intellectual disabilities.	OHRQoL was lower among children classified in the high dental caries severity group, with 81.2% of children with ASD falling into this category.
Qiao et al., 2020, China [58]	To evaluate oral and behavioural problems and their impact on the OHRQoL of children with ASD compared with typically developing children.	Cross-sectional study	144 children with ASD and 228 children with typically developing children ages: 3–16 years	A self-administered questionnaire collecting sociodemographic information and oral health habits; Modified version of the P-CPQ; FIS	Children with ASD presented significantly more oral symptoms than typically developing children (99.2% vs. 94.1%, *p* < 0.001). Statistically significant differences were also observed between the groups (*p* < 0.005) in the prevalence of halitosis, oral lesions, pain, mouth breathing, and object biting. In addition, 39.6% of children with ASD brushed their teeth twice daily, whereas 51.8% had never visited a dental clinic. Parents of children with ASD reported higher scores across all domains of the P-CPQ.	ASD was associated with poorer OHRQoL for both ASD children and their families, particularly among older children.
Saini et al., Saudi Arabia, 2022 [59]	To evaluate the influence of sociodemographic factors on parents’ perceptions of OHRQoL of their children with ASD.	Cross-sectional study	100 parents with children with ASD, aged 4–15 years	P-CPQ; A self-administered questionnaire was used to collect sociodemographic data, including parents’ age, family income, and educational level	Parents’ age and educational level were significantly associated with the oral symptoms and emotional well-being domains of the P-CPQ (*p* < 0.05). Family income was significantly associated with the oral symptoms domain (*p* < 0.001).	Parents’ age, educational level, and family income influenced their perceptions and their ability to care for their children with ASD.
Salerno et al., Italy, 2024 [60]	To evaluate the OHRQoL of children with ASD who followed a preventive program compared with children with ASD who did not receive preventive programme and with typically developing children.	Comparative cross-sectional study	A total of 909 parents: 168 parents of children with ASD enrolled in a preventive programme, 168 parents of children with ASD not enrolled in a preventive programme, and 336 parents of typically developing children.	P-CPQ; FIS; A self-administered questionnaire was used to collect data on the children’s age and the parents’ demographic and socioeconomic characteristics	Parents of children with ASD reported poorer OHRQoL than parents of typically developing children. The only P-CPQ domain that differed between the two ASD groups was emotional well-being. Significant differences were also found between the two ASD groups in the Family Impact Scale (FIS) domains of Emotions and Conflict, with lower scores observed among children enrolled in the preventive programme. No significant differences were found between the groups in the oral symptoms domain.	Regular preventive oral care had a positive impact on the OHRQoL of children with ASD. In addition, it was associated with reduced parental stress and improved overall family quality of life.
Scarpis et al., Italy, 2026 [61]	To evaluate parents’ and caregivers’ perceptions of the impact of oral conditions on OHRQoL of children and adolescents with ASD compared with children and adolescents without ASD.	Comparative cross-sectional study	Test group: 31 parents of children with ASD, aged 6–14 years. Control group: 51 parents of typically developing children, aged 6–14 years.	P-CPQ	The overall P-CPQ scores for children with ASD were significantly higher than those for typically developing children (31.0 vs. 10.6, *p* < 0.0001). Children with ASD also had significantly higher scores in the functional limitations (6.9 vs. 1.0), emotional well-being (7.0 vs. 4.0), and social well-being (11.1 vs. 1.0) domains. However, no significant differences were observed in the oral symptoms domain, where both groups had a median score of 3.0.	Parents of children and adolescents with ASD perceived their children’s OHRQoL as poorer than did parents of typically developing children. In some cases, the impact of oral conditions may be overlooked because parents of children with ASD are heavily burdened by the child’s overall health needs.
Veseli et al., 2026, Kosovo [62]	To evaluate parent- and caregiver-reported differences in OHRQoL between children with ASD and typically developing children.	Comparative cross-sectional study	Test group: 40 children with ASD, aged 4–6 years, and their parents Control group: 40 children without ASD, 4–6 years, and their parents	World Health Organization questionnaire with 10 additional questions specific to ASD; P-CPQ	Oral health status was assessed using the dmft index, the Gingival Index, and the Plaque Index. The WHO questionnaire also collected information on the reasons for dental visits and self-injurious behaviours. A total of 86.7% of parents of children with ASD reported that dental visits were difficult, compared with only 28.9% of parents in the control group (*p* < 0.001). No significant differences were found in oral health status between the two groups.	Parents of children with ASD reported significantly higher scores for emotional well-being, functional limitations, and social well-being.
Viana et al., Brazil, 2022 [63]	To evaluate the impact of oral conditions on the OHRQoL of children and adolescents with ASD from the perspective of their parents/caregivers.	Cross-sectional study	40 children and adolescents with ASD (aged 6–14 years) receiving care at seven institutions for individuals with special healthcare needs.	P-CPQ; FIS	A total of 97.5% of mothers reported that oral conditions had an impact on their children’s quality of life. A higher caregiver educational level, measured by years of schooling, was associated with a more favourable perception of their child’s quality of life (*p* = 0.016). Most children and adolescents with ASD had no history of dental caries and exhibited good to moderate oral hygiene.	The study suggests that parents’ and caregivers’ educational level plays an important role in the OHRQoL of children and adolescents with ASD. No significant differences in OHRQoL scores were found according to oral hygiene status or the prevalence of dental caries.

### 3.2. OHRQoL Tool

Among the 23 studies included in this review, 15 used the P-CPQ Questionnaire [42,43,45,46,48,49,51,56,57,58,59,60,61,62], making it the most frequently employed instrument for assessing oral health-related quality of life (OHRQoL). The FIS Questionnaire was used in nine studies [42,43,46,47,48,51,58,60,63], most commonly in combination with the P-CPQ to evaluate the impact of children’s oral health on family functioning.

Three studies employed the PedsQL™ 4.0 Questionnaire [44,52,53], while the ECOHIS Questionnaire was used in two studies [50,52]. In addition, several authors used specific assessment instruments, including the FHC-OHRQoLQuestionnaire [41], the BREALD-30 Questionnaire [46], and the EQ-5D-Y Questionnaire [54]. Furthermore, Prakash et al. [55] developed a seven-item questionnaire to assess parents’ knowledge, attitudes, and perceptions regarding the OHRQoL of children with ASD.

The analysis of the included studies indicates that the majority of parents of children with autism spectrum disorder (ASD) perceived their children’s OHRQoL to be poorer than that of neurotypical children. The factors most frequently associated with impaired OHRQoL included dental caries, dental pain, bruxism, functional limitations, and difficulties in accessing dental care. Many studies also reported a substantial impact on families, manifested by increased parental stress, emotional burden, and limitations in daily activities. Parental educational attainment, household income, and oral health literacy were identified as significant determinants of perceived OHRQoL in several studies. Conversely, dental treatment and regular preventive programmes were consistently associated with significant improvements in OHRQoL for both children and their families. Although a small number of studies did not identify significant differences between children with and without ASD in certain OHRQoL domains, the overall body of evidence supports a greater negative impact of oral conditions on children with ASD. Collectively, these findings highlight the need to implement targeted preventive programmes and provide dental services tailored to the specific needs of this population, while also supporting parents and caregivers through educational initiatives and multidisciplinary care.

**Figure 3:**
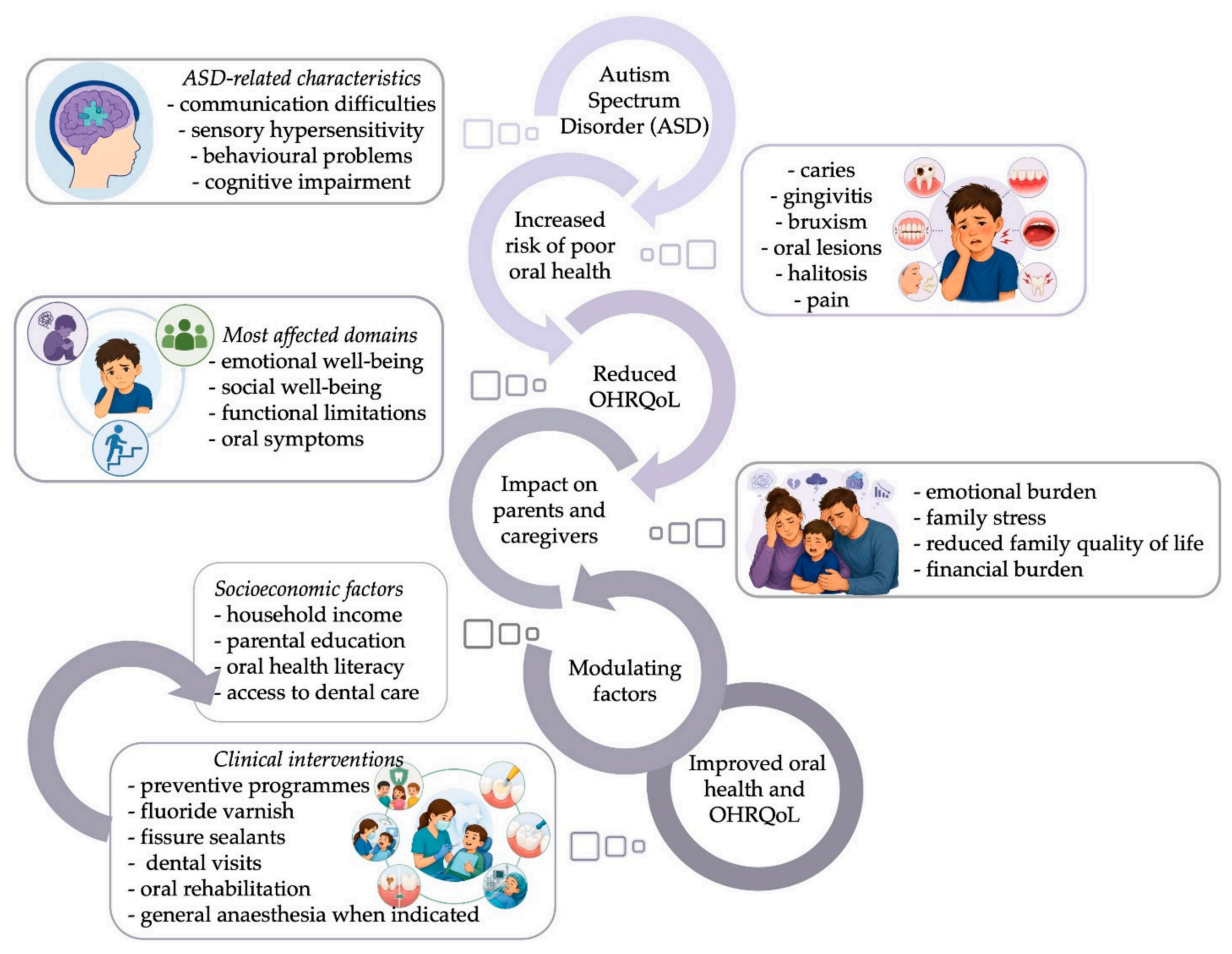
OHRQoL in children and adolescents with ASD.

Figure 3 summarises the effects of oral health status on the quality of life of children and adolescents with ASD and their families.

## 4. Discussion

To critically appraise the selected studies, several factors potentially influencing the OHRQoL of children and adolescents with ASD were analysed. These included oral health status and clinical manifestations, preventive and restorative dental treatment, socio-demographic and socioeconomic factors such as caregivers’ age, oral health literacy, household income, and educational attainment. In addition, the questionnaires used in the included studies and their suitability and validity for the respective research objectives were reviewed.

### 4.1. OHRQoL Assessment Instruments

The oral cavity plays an essential role in fundamental human functions, including mastication, speech, respiration, facial aesthetics, and emotional expression. The most common oral diseases affecting children and adolescents, namely dental caries and gingivitis, may compromise oral health and function, thereby negatively affecting multiple dimensions of OHRQoL, as well as physical development, mental health, and socio-emotional well-being [64,65].

Several validated instruments have been developed to assess OHRQoL in children and adolescents, including the Child Perceptions Questionnaire (CPQ), the Child Oral Health Impact Profile (COHIP), the Child Oral Impacts on Daily Performances (C-OIDP), the Early Childhood Oral Health Impact Scale (ECOHIS), and the Pediatric Quality of Life Inventory (PedsQL™) [36].

For children with ASD, who often experience cognitive impairments that limit their ability to complete self-report questionnaires, OHRQoL assessment relies primarily on parents’ or caregivers’ perceptions. One of the most widely used proxy-report instruments is the P-CPQ, developed by Jokovic et al. in 2003 [66].

The P-CPQ consists of 16 items assessing children’s oral health and its impact on overall well-being across four domains: oral symptoms, functional limitations, emotional well-being, and social well-being. Responses are recorded using a five-point Likert scale, with scores ranging from 0 (“don’t know”) to 5 (“every day or almost every day”). The total P-CPQ score ranges from 0 to 80, with higher scores indicating poorer OHRQoL [51].

Another widely used proxy-report instrument is the Family Impact Scale (FIS), developed by Locker et al. in 2002 [67]. The FIS comprises eight items assessing the impact of children’s oral health on family quality of life across three domains: family activities, parental emotions, and family conflict. Total scores range from 0 to 40, using the same response options as the P-CPQ. As with the P-CPQ, higher scores indicate poorer family quality of life. Both instruments have demonstrated good validity for assessing OHRQoL in children with special healthcare needs, although cross-cultural adaptation and linguistic validation are required before use in different populations.

The studies included in this review consistently reported higher parental P-CPQ scores for children with ASD, reflecting poorer perceived OHRQoL. For example, de Souza et al. [51] found that Brazilian children and adolescents with ASD experienced the greatest impact in the functional limitations domain of the P-CPQ. Similar findings were reported by Qiao et al. [58], whose study showed significantly higher parental scores across all P-CPQ domains in children with ASD. Likewise, Veseli et al. [62] observed significantly higher scores for emotional well-being, functional limitations, and social well-being, while Scarpis et al. [61] reported significantly higher scores among children with ASD than among neurotypical children in the domains of functional limitations (6.9 vs. 1.0), emotional well-being (7.0 vs. 4.0), and social well-being (11.1 vs. 1.0).

Using the FIS Questionnaire, da Silva et al. [47] demonstrated that parents of children with ASD experienced a greater emotional burden, which was associated with responsibility for toothbrushing. This burden was also associated with the need for orthodontic treatment and household income.

Comparable findings were reported by Eslami et al. [53], who used the PedsQL Oral Health Scale and found that children with ASD experienced significantly more difficulties in the social (*p* = 0.027) and communication (*p* = 0.004) domains than neurotypical controls.

### 4.2. OHRQoL and Oral Health Status

Children with ASD frequently experience difficulties in maintaining good oral health because of selective dietary habits, self-injurious oral behaviours, and barriers to accessing dental care. They commonly present poorer oral hygiene, higher rates of periodontal disease and dental caries, and an increased prevalence of oral trauma, all of which may negatively influence OHRQoL and highlight the complex oral healthcare challenges faced by this population [29,68,69,70].

The included studies consistently reported a high prevalence of oral diseases among children with ASD. In Brazil, Carrada et al. [46] found that 40% of children had at least one decayed tooth and 36.7% presented untreated caries. In Croatia, Badrov et al. [43] reported prevalences of 48.8% for both dental caries and dental plaque, 38.8% for bruxism, and 31.4% for dental calculus. In China, Qiao et al. [58] observed prevalences of 56.4% for dental caries, 62.5% for oral lesions, and 95.6% for halitosis. In India, Prakash et al. [55] found that 59.1% of children with ASD had dental caries. Similarly, in Saudi Arabia, Alaki et al. [41] reported significantly more extraoral lesions (traumatic injuries or scars), intraoral lesions, a higher prevalence of dental caries (*p* = 0.013), and greater caries severity (*p* = 0.003) in children with ASD than in healthy controls.

Several additional studies confirmed the association between poor oral health and impaired OHRQoL. Procopio et al. [56] demonstrated that dental caries experience was associated with a significantly greater negative impact on OHRQoL among children with ASD (*p* < 0.05). Likewise, de Souza et al. [51] reported that caries experience in the primary dentition was associated with higher scores across all P-CPQ domains. Puthiyapurayil et al. [57], in a study involving children with intellectual disabilities, concluded that children with severe dental caries had poorer OHRQoL; notably, 81.2% of children with ASD belonged to the highest caries severity category and exhibited the highest P-CPQ scores among all disability groups. Fallea et al. [54] identified a positive association between oral health status assessed using OHAT and quality of life measured by the EQ-5D-Y (*p* = 0.00271). Furthermore, Du et al. [52] evaluated both oral and general health, reporting that the risk of poor OHRQoL was fourfold higher among children with previous dental treatment experiences (*p* < 0.001) and 3.2-fold higher in relation to the child’s general medical condition (*p* < 0.001).

Nevertheless, poorer oral health among children with ASD does not invariably translate into poorer OHRQoL. Veseli et al. [62], Viana et al. [63], and Carrada et al. [46] found no statistically significant association between oral health status and OHRQoL. Similarly, Cancio et al. [45] reported significantly greater impacts within the social well-being domain among children without caries experience (*p* < 0.05).

The contradictory findings reported across the included studies may be explained by several factors that were not adequately addressed. First, the severity of ASD was not specified, despite being an important determinant of a child’s cognitive functioning. Consequently, it is difficult to establish whether children were able to maintain adequate oral hygiene practices or effectively communicate symptoms such as pain and discomfort to their parents or caregivers.

Second, several studies did not report essential information regarding parents or caregivers, including their level of oral health literacy, educational attainment, socioeconomic status, and their role in managing the child’s behaviour. These factors may substantially influence both oral health practices and parental perceptions of the child’s oral health-related quality of life.

Finally, differences in the reported OHRQoL outcomes may also be attributable to variations in families’ access to dental care services and the level of cooperation established among children with ASD, their families, and paediatric dentists during dental treatment. These factors may significantly influence both the oral health status of children with ASD and the perceptions reported by their parents or caregivers.

### 4.3. OHRQoL and Dental Treatment

Children with ASD frequently experience difficulties in receiving dental treatment because of behavioural challenges, sensory hypersensitivity, communication impairments, and limited understanding of dental procedures. Owing to sensory hypersensitivity, psychomotor agitation, behavioural distress, and difficulties in cooperating during treatment, many children with ASD require conscious sedation or general anaesthesia to undergo dental procedures. This increases both the complexity and the cost of care. In this context, preventive programmes and comprehensive oral rehabilitation have the potential to improve the oral health-related quality of life (OHRQoL) of these patients [30,61,71,72,73].

Within this context, Al Aswad et al. [42] conducted a study in Saudi Arabia to evaluate parents’ perceptions of the OHRQoL of children with and without ASD following comprehensive oral rehabilitation under general anaesthesia. The authors concluded that both groups achieved comparable P-CPQ scores, indicating that comprehensive oral rehabilitation under general anaesthesia had a positive impact on OHRQoL in both children with ASD and their neurotypical peers.

Similarly, Salerno et al. [60] evaluated the OHRQoL of children with ASD who participated in a preventive oral healthcare programme, comparing them with children with ASD who did not receive preventive care and with neurotypical children. Preventive interventions included the application of 5% sodium fluoride varnish and fissure sealants, while restorative treatment focused on the management of dental caries. The study found no statistically significant differences between the ASD groups in the oral symptoms domain of the P-CPQ. Significant differences were observed only in the functional limitations, emotional well-being, and social well-being domains. These findings may be explained by reduced parental stress and improvements in the overall quality of family life.

Comparable objectives were addressed by de Paula et al. [50] in Brazil, who used the B-ECOHIS questionnaire to assess the OHRQoL of children with ASD before dental treatment and 14 days after treatment. The authors reported reductions in both the overall B-ECOHIS score and all domain scores following treatment, indicating an improvement in OHRQoL. Likewise, de Almeida et al. [49], also in Brazil, used the P-CPQ and observed a significant reduction in parental perception scores three months after dental treatment, with mean scores decreasing from 13.2 ± 6.4 to 3.4 ± 2.2 (*p* < 0.001).

### 4.4. OHRQoL and Socio-Demographic Factors

Several socio-demographic factors have been identified as determinants of OHRQoL in children and adolescents, including those with special healthcare needs. These include lower parental educational attainment, lower household income, and limited parental oral health literacy [74,75].

The studies included in this review demonstrated significant associations between parental age and educational level and specific domains of the P-CPQ, particularly oral symptoms and emotional well-being [47,48,59]. The greater emotional burden reported by caregivers of children with ASD may be explained by multiple factors, including the child’s level of dependence and parenting responsibilities. It may also be explained by challenges in accessing both oral and general healthcare services. Together, these factors contribute to the increased emotional stress experienced by many caregivers [47].

With regard to household income, the available evidence is inconsistent. de Souza et al. [51] found no significant association between household income and OHRQoL scores. In contrast, Carrada et al. [46] reported that families with a monthly income of up to two minimum wages were 2.25 times more likely to perceive their children’s oral health-related quality of life negatively than families with a higher household income.

### 4.5. OHRQoL and Parental Oral Health Literacy

Parental oral health literacy plays a fundamental role in maintaining children’s oral health and improving their quality of life by promoting appropriate oral hygiene practices and encouraging positive attitudes towards dental attendance. It also facilitates the adoption of preventive oral healthcare measures, thereby contributing to improved oral health outcomes and quality of life [76,77,78].

The association between parental oral health literacy and children’s OHRQoL remains inconsistent in the literature. While some studies have demonstrated a significant relationship, others have reported weak or non-significant associations [79,80].

For parents of children with ASD, these challenges are even greater because of communication difficulties, behavioural disturbances, and the practical challenges associated with performing daily oral hygiene procedures [44].

Limited parental or caregiver knowledge regarding the characteristics of ASD and common childhood oral diseases may contribute to the neglect of children’s oral healthcare needs [51]. Insufficient access to specialised behavioural therapies or appropriate dental treatment may also contribute to the neglect of these needs.

These challenges are compounded by the communication impairments often exhibited by children with ASD, which may prevent parents from recognising pain and discomfort associated with oral disease, ultimately affecting the quality of life of the entire family.

In their study conducted in Türkiye, Beyaz and Öznurhan [44] found statistically significant differences between parents of children with ASD and parents of neurotypical children only in the social functioning domain and in the overall POQL score. Parents in the control group also achieved significantly higher scores on the oral health literacy assessment than parents of children with ASD, suggesting that lower parental oral health literacy may contribute to poorer perceived OHRQoL among children with ASD.

Supporting children with ASD requires the implementation of targeted strategies, including early screening for the condition, parental education programmes, and the active involvement of oral healthcare and mental healthcare professionals in multidisciplinary care [81].

Within oral healthcare, technology-based interventions, including electronic media and digital applications, have shown considerable promise in supporting oral health education and improving dental care delivery for this population [82]. One such example is the study by Lefer et al. [83], who implemented a tablet-based training programme using pictograms to teach toothbrushing skills. Their findings demonstrated that the application improved toothbrushing performance and promoted greater independence in daily oral hygiene routines.

Another noteworthy initiative, aimed at both parents and dental professionals, was developed by Tan et al. [84], who designed a mobile application incorporating social stories, visual schedules, and direct communication tools for caregivers and dentists. Their results indicated that the application was well accepted and enhanced users’ awareness of, and access to, oral healthcare resources.

Future research should focus on the development of standardised clinical guidelines, the implementation of parental education programmes, improving access to dentists trained in the management of children with ASD, and the evaluation of innovative approaches, such as digital interventions, to optimise oral healthcare delivery for this population [82].

### 4.6. Gaps in Literature and Future Research Challenges

The present scoping review identified several gaps in the existing literature, indicating the need for further research to provide a more comprehensive understanding of this topic. The main limitations identified across the included studies include the lack of longitudinal studies assessing the oral health-related quality of life of children with ASD over medium- and long-term follow-up periods, as well as the scarcity of interventional studies investigating the effectiveness of innovative, condition-specific treatment approaches. Furthermore, the included studies showed considerable heterogeneity regarding the severity of ASD and the degree of neurological impairment, both of which are important factors that may account for variations in the behavioural characteristics of children with ASD. Another important limitation concerns the assessment instruments, as only two standardised questionnaires, the P-CPQ and the FIS, have been specifically validated for children with reduced cognitive abilities, whereas several studies developed their own questionnaires, potentially contributing to inconsistent and non-comparable findings. Finally, there is a need for large multicentre studies including representative samples of children with ASD from regions with different levels of socioeconomic development to improve the generalisability of the available evidence.

### 4.7. Implications for Clinical Practice 

The findings of this scoping review have important implications for paediatric dental practitioners by providing a comprehensive understanding not only of the oral health needs of children with ASD but also of parents’ and caregivers’ perceptions of the quality of life of families caring for these children, as well as the emotional and financial burden associated with meeting their oral healthcare needs.

Furthermore, this review highlights existing gaps in the education and support provided to parents and caregivers of children with special healthcare needs. Strengthening collaboration between dentists, psychologists, and families is essential for maintaining and improving the oral health-related quality of life and overall well-being of children and adolescents with ASD.

### 4.8. Limitations of the Review

This review has several limitations. Firstly, although the search strategy was designed to be as comprehensive as possible, some relevant studies may have been missed. Secondly, only studies published between January 2016 and May 2026 and written in English were included, resulting in a total of 23 eligible studies. This relatively small number limits the generalisability of the findings. Another limitation is that most of the included studies employed a cross-sectional design, which precludes the establishment of causal relationships between oral health status and oral health-related quality of life.

Geographical representation also constitutes a limitation, as ten of the included studies were conducted in Brazil, potentially leading to findings that predominantly reflect the social, cultural, and economic context of a single country. Furthermore, because this review focused on parent- or caregiver-reported OHRQoL, caregivers may not always accurately recognise or estimate the impact of oral symptoms on their children. Their perceptions may also be influenced by factors such as family stress or the severity of the child’s autism spectrum disorder.

Finally, the considerable heterogeneity of the questionnaires used across the included studies limits direct comparisons between studies and reduces the overall comparability of the reported findings.

## 5. Conclusions

The Parental-Caregiver Perceptions Questionnaire was the most frequently used instrument for assessing oral health-related quality of life. Across the majority of the included studies, children with ASD obtained significantly higher P-CPQ scores than their neurotypical peers, indicating poorer oral health-related quality of life. The evidence synthesised in this review indicates that children and adolescents with ASD generally experience poorer OHRQoL than neurotypical children. This reduced quality of life appears to be associated with compromised oral health status, limited access to specialised dental care, and inadequate oral hygiene practices. The domains most frequently identified by parents as being adversely affected were social well-being and emotional well-being, followed by functional limitations and oral symptoms. Parents and caregivers consistently reported that oral conditions, including dental caries, gingivitis, halitosis, oral lesions, and dental trauma, contributed substantially to discomfort among children with ASD, while the increased oral healthcare needs imposed considerable emotional and practical burdens on the entire family. In addition, lower household income and limited parental oral health literacy were identified as important determinants of parents’ negative perceptions of both their children’s and their families’ oral health-related quality of life. Conversely, preventive dental interventions and comprehensive oral rehabilitation performed under general anaesthesia were consistently associated with improvements in OHRQoL. Overall, these findings emphasise the need to implement educational and counselling programmes for parents and caregivers of children with ASD, enabling them to better manage the daily challenges associated with providing oral care and supporting the overall well-being of their children.

## Figures and Tables

**Table 1:** Search strategies.

Database Name	Search Terms	Number of Articles Found
PubMed/Medline	autism OR autism spectrum disorder OR autistic disorder AND child OR adolescent AND oral health OR oral health-related quality of life OR OHRQoL AND parental perception OR parent perception	144
Scopus	autism OR autism spectrum disorder OR autistic disorder AND child OR adolescent AND oral health OR oral health-related quality of life OR OHRQoL AND parental perception OR parental attitude	82
Web of science	oral health-related quality of life AND autism spectrum disorder OR oral health-related quality of life AND autism AND children AND adolescents OR autism spectrum disorder AND parental perception AND oral health	131
Embase	autism AND oral health-related quality of life OR autism spectrum disorder AND oral health quality of life AND children AND adolescents OR autism spectrum disorder AND oral health-related quality of life OR autism AND oral health-related quality of life AND children AND adolescents OR autism spectrum disorder AND parental perception	110
Google Scholar	autism spectrum disorder OR autism OR autistic disorder AND oral health-related quality of life OR oral health AND (children OR adolescents) AND parental perception OR parents	332

**Table 2:** Inclusion and exclusion criterion.

Criterion	Inclusion	Exclusion
Type of study	Cross-sectional studies, analytical studies	Systematic review, narrative review, scoping review
Participants	Parents, children and adolescents with ASD	Children and adolescents with other disabilities: cerebral palsy, attention deficit hyperactivity disorder, intellectual disability, visual and hearing impairment, Down syndrome
Evaluation period	Articles published between 2016–May 2026	Articles published before 2016
Accessibility	Full-text articles in English, with open access or access through the university	Articles in abstracts or not freely accessible, articles published in other languages than English
Intervention	Studies that aim to evaluate the OHQRoL of children and adolescents with ASD	Irrelevant articles, which do not correspond to the proposed purpose, or which do not have a clear research methodology

**Table 3:** Data extraction template.

Category	Details
General Information	The first author of the article, year of publication, country and region where the research was conducted
Study design	Cross-sectional studies/analytical studies
Study objective and aim	The main objectives of the research are to evaluate the OHRQoL of children and adolescents with ASD and to evaluate the parental perception and perspectives
Population	Sampling method, number of subjects, age
Intervention	Type of questionnaire used, other types of interventions: oral examination, dental procedures
Key findings and conclusions	Main findings, conclusions, future research suggestions, limitations

## Data Availability

The original contributions presented in this study are included in the article and supplementary material. Further inquiries can be directed to the corresponding author.

## Supplementary Materials

The following supporting information can be downloaded at: https://www.mdpi.com/article/10.3390/dj14080492/s1. Table S1: Preferred Reporting Items for Systematic reviews and Meta-Analyses extension for Scoping Reviews (PRISMA-ScR) Checklist.

## Abbreviations

The following abbreviations are used in this manuscript:

ASD	Autism Spectrum Disorder
IOTN	Index Of Orthodontic Treatment Need
OHRQoL	Oral Health-Related Quality-of-Life
HRQoL	Health-Related Quality-of-Life
P-CPQ	Parental-Caregiver Perceptions Questionnaire
FIS	Family Impact Scale
OHAT	Oral Health Assessment Tool
POQL	Pediatric Oral Health-Related Quality of Life
B-ECOHIS	Brazilian Early Childhood Oral Health Impact Scale
EQ-5D-Y	Euroqol 5-Dimensions Youth Version
TOHLAT-P	Oral Health Literacy Assessment Task for Pediatric Dentistry
PedsQL	Pediatric Quality-of-Life Inventory
ECOHIS	Early Childhood Oral Health Impact Scale
FHC-OHRQoL	Franciscan Hospital for Children Oral Health-Related Quality of Life Questionnaire
BREALD-30	Brazilian Version of the Rapid Estimate of Adult Literacy in Dentistry
